# Traveling Subject-Informed Harmonization Increases Reliability of Brain Diffusion Tensor and Neurite Mapping

**DOI:** 10.14336/AD.2023.1020

**Published:** 2023-11-15

**Authors:** Yuya Saito, Koji Kamagata, Christina Andica, Norihide Maikusa, Wataru Uchida, Kaito Takabayashi, Seina Yoshida, Akifumi Hagiwara, Shohei Fujita, Toshiaki Akashi, Akihiko Wada, Ryusuke Irie, Keigo Shimoji, Masaaki Hori, Kouhei Kamiya, Shinsuke Koike, Takuya Hayashi, Shigeki Aoki

**Affiliations:** ^1^Department of Radiology, Juntendo University Graduate School of Medicine, Tokyo Japan.; ^2^Faculty of Health Data Science, Juntendo University, Chiba, Japan.; ^3^Center for Evolutionary Cognitive Sciences, Graduate School of Art and Sciences, The University of Tokyo, Tokyo, Japan.; ^4^Department of Radiological Sciences, Graduate School of Human Health Sciences, Tokyo Metropolitan University, Tokyo, Japan.; ^5^Department of Radiology, The University of Tokyo, Tokyo, Japan.; ^6^Department of Radiology, Toho University Omori Medical Center, Tokyo Japan.; ^7^Laboratory for Brain Connectomics Imaging, RIKEN Center for Biosystems Dynamics Research, Japan.; ^8^Department of Brain Connectomics, Kyoto University Graduate School of Medicine

**Keywords:** multisite study, harmonization, diffusion magnetic resonance imaging, diffusion tensor imaging, neurite orientation dispersion, and density imaging

## Abstract

Diffusion-weighted magnetic resonance imaging (dMRI) of brain has helped elucidate the microstructural changes of psychiatric and neurodegenerative disorders. Inconsistency between MRI models has hampered clinical application of dMRI-based metrics. Using harmonized dMRI data of 300 scans from 69 traveling subjects (TS) scanning the same individuals at multiple conditions with 13 MRI models and 2 protocols, the widely-used metrics such as diffusion tensor imaging (DTI) and neurite orientation dispersion and density imaging (NODDI) were evaluated before and after harmonization with a combined association test (ComBat) or TS-based general linear model (TS-GLM). Results showed that both ComBat and TS-GLM significantly reduced the effects of the MRI site, model, and protocol for diffusion metrics while maintaining the intersubject biological effects. The harmonization power of TS-GLM based on TS data model is more powerful than that of ComBat. In conclusion, our research demonstrated that although ComBat and TS-GLM harmonization approaches were effective at reducing the scanner effects of the site, model, and protocol for DTI and NODDI metrics in WM, they exhibited high retainability of biological effects. Therefore, we suggest that, after harmonizing DTI and NODDI metrics, a multisite study with large cohorts can accurately detect small pathological changes by retaining pathological effects.

## INTRODUCTION

Neural wiring allows functional ensembles, subserves cognitive and emotional behaviors, and constitutes the white matter (WM) disproportionately enlarged in humans than in other mammals [1, 2]. Diffusion-weighted magnetic resonance imaging (dMRI) is a powerful noninvasive tool that provides information on water molecule diffusion property. A classical model for interpreting dMRI signals is diffusion tensor imaging (DTI) that describes the Gaussian properties of diffusion motion by an algebraic object, tensor, indirectly associated with nerve fiber tract organization and myelination [3]. Recently, a more sophisticated biophysical model for dMRI, neurite orientation dispersion and density imaging (NODDI), has gained attention to infer the WM organization more specific to neurites [4]. DTI and NODDI have successfully advanced our understanding of the integrity of human brain tissue microstructure in development [5], plasticity [6], aging [7], psychiatric [8] neurologic disorders [9], and mental conditions [10, 11]; however, values of dMRI in medical diagnosis is limited particularly for assessing mental health and neuropsychiatric disorders.

One of reasons for limited utility of dMRI in mental health may be small effect size and inconsistent results across studies [12-15]. For instance, the DTI effect sizes for bipolar disorder were 0.020 - 0.050 (R squared) in body of corpus callosum (BCC), 0.015 - 0.038 in cingulate gyrus part of the cingulum (CGC), and 0.010 - 0.030 in fornix [13]. Additionally, the DTI effect sizes of schizophrenia were -2.00 - 1.00 (Cohen’s *d*) in BCC, -1.50 - 1.50 in CGC, and -1.50 - 1.00 in fornix [14]. Thus, there are large differences in effect size or even opposite effect between studies. Inconsistency between studies may be ascribed to sample size limitation and lack of statistical power, as proven by the small sample size (approximately 25) in most neuroimaging studies [16]. Recent studies targeting mental health indeed suggest a need for a sample size of at least several thousand people to obtain reproducible results [16]. Multisite population studies with large datasets, including dMRI, have been recently conducted in healthy participants in the Human Connectome Project (HCP) from 1200 participants [17], and UK Biobank from 100,000 participants [18]. However, collecting data from different sites undoubtedly biases measurements due to differences in MRI systems, such as hardware (e.g., inhomogeneities of B0 field, B1 transmitter, and receive fields), acquisition parameters (e.g., echo time, repetition time, *b*-value scheme, diffusion time, number of diffusion gradient directions, and voxel size), reconstruction algorithms, which can lead to variability in signal-to-noise ratio (SNR) [19], reproducibility, and bias to detect disease-related signal changes [13, 14]. Indeed, reproducibility of DTI and NODDI measures was degraded when used different MRI sites, models, and protocols [4, 20-24]. Close inspection of microstructures is expected to increase sensitivity to changes in the brain wiring and function but requires large data acquisitions, preprocessing, and statistics [25].

In the last decade, novel statistical approaches have been developed to harmonize neuroimaging-based metrics acquired in different MRI models or protocols. The first attempt to utilize sites as covariates in a general linear model (GLM) eliminated measurement bias (scanner effect) as well as biologically meaningful sampling biases (subject effect) [26]. Recently, the combined association test (ComBat), an extension of GLM with an empirical Bayesian-based harmonization, was developed to specifically eliminate the only measurement bias maintaining sampling bias [27]. ComBat was indeed proven useful for harmonizing fractional anisotropy (FA) and mean diffusivity (MD) acquired from multisite DTI data and increasing statistical power [28]. However, ComBat may still be insufficient to accurately discriminate measurement bias from scanner effects or sampling bias from true group-wise biological differences in the statistical design [29]. Therefore, the traveling subjects GLM (TS-GLM) is a recently proposed harmonization method using TS datasets that has worked well for functional connectivity using resting-state functional MRI (rs-fMRI) [29] and gray matter volumetry via T1-weighted structural MRI [30]. However, the performance of TS-GLM for harmonizing diffusion metrics has not been well established.

This study aimed to validate harmonization methods for harmonizing DTI and NODDI metrics in WM areas using large-scale TS subject data collected in the Brain/MINDS Beyond Human Brain MRI project (BMB-HBM) (hbm.brainminds-beyond.jp) [25]. The BMB-HBM multisite TS dataset was obtained by different factors of MRI measurements in the largest number of TS (69 participants, 13 sites, 7 models, and 2 protocols, resulting in a total of 245 scans) among others [31-33], including multi-modal MRI datasets, such as high-resolution T1-weighted, T2-weighted structural MRI, multi-shell dMRI, and resting-state fMRI. Of these, dMRI data were collected in two protocols: a BMB-HBM harmonized protocol (HARP) that allows HCP-like high-quality scanning [25] and HCP protocol for Connectomes Related to Human Disease (CRHD) [34]. In the dataset, scan-rescan data were used to evaluate measurement bias and harmonization performance following the same procedure. We evaluated the performance of TS-GLM, as well as ComBat for statistically harmonizing dMRI metrics between MRI sites, models, and protocols. Additionally, we assessed the ability of the harmonization methods to retain neurobiological changes related to age and sex.

## MATERIALS AND METHODS

### Study cohorts

The local Ethics Committee approved this study, and all participants provided written informed consent. The TS subject project (75 participants, 13 sites, 15 models, two protocols, a total of ~450 scans) adopted a “hub-and-spoke” design (each participant traveled to five or six sites but not all sites: UTK, The University of Tokyo ECS (Komaba Campus); UTI, The University of Tokyo IRCN; FUM, Fukushima Medical University; TMG, Tamagawa Academy & University; SWA, Showa University; NCNP, National Center of Neurology and Psychiatry; JTD, Juntendo Hospital; ATR, Advanced Telecommunications Research Institute International; UOS, Osaka University; UHI, Hiroshima University; UNG, Nagoya University; UKY, Kyoto University; KRC, Kyoto University Kokoro Research Center) [25]. Subsequently, we selected data (69 participants, 13 sites, seven models, and two protocols, a total of 245 scans) from the TS subject project data and assigned it to four datasets according to the measurement biases of site, model, protocol (Table 1). In the dataset, 55 scan-rescan brain images were also included using the site, model, and protocol in their recruited sites and the obtained rescan data were also used for confirming the stability of the DWI values. All participants were assessed for any brain abnormalities, including healthy participants. Although the 3T MRI contained 7 Siemens models: Prisma, Prisma fit, Skyra, Skyra fit, Verio, Verio dot, and Trio, the dataset of between-site consisted of five sites (ATR, JTD, UOS, UTI, and UTK) and a single model and protocol. Moreover, between-model dataset consisted of five models (Prisma, Prisma fit, Skyra, Verio, and Verio dot), a single site, and a protocol. The dataset of between-protocol also consisted of two protocols, a single site, and a model. This process was how the scanner effects were separated into between-site, -model, and -protocol, followed by an evaluation of harmonization performance in detail.

### MRI acquisition

Each MRI acquisition was performed using a 32-channel head coil and obtained dMRI with a single-shot spin-echo-planar sequence and monopolar diffusion gradient. The MRI acquisition parameters are shown for each protocol in Table 2. To correct geometric distortion due to susceptibility artifacts, dMRI data were acquired with phase encoding in the A-P and P-A directions. For procedure 1, the following parameters were held constant across models; *b* = 0/700/2000 s/mm^2^, repetition time = 3600 ms, flip angle 90°, 1.7 mm isotropic voxel, 84 axial slices, simultaneous multislice acquisition with acceleration factor 3, and partial Fourier 6/8. Conversely, for procedure 2, *b* = 0/1500/3000 s/mm^2^, repetition time = 3230 ms, flip angle 78°, 1.5 mm isotropic voxel, 92 axial slices, simultaneous multislice acquisition with acceleration factor 4, and partial Fourier 6/8.

### dMRI processing

Acquired dMRI data were preprocessed using FSL 6.0.1 and MRTrix3, following recent studies on optimal preprocessing [35]. First, MR magnitude images were denoised using Marchenko-Pastur principal component analysis (MP-PCA) as a denoise algorithm [36] and corrected for Gibbs artifacts [37]. Next, we adopted an analytical approach and used the noise standard deviation estimated by applying MP-PCA on the lower *b*-value (Procedure 1, *b* = 0, 700 s/mm^2^; Procedure 2, *b* = 0, 1500 s/mm^2^) to reduce possible biases caused by Rician noise distribution. Additionally, the effect of eddy currents and motion, including dMR images, were corrected using the “*topup*” and “*eddy*” commands in the FSL software and B1 inhomogeneity. Then, the resulting dMR images were fitted to the NODDI model Toolbox 5 (www.nitrc.org/projects/noddi_toolbox) to generate (i.e., NDI, ISOVF, and ODI maps using three *b*-values (Procedure 1, *b* = 0, 700, 2000 s/mm^2^; Procedure 2, *b* = 0, 1500, 3000 s/mm^2^). Later, while the diffusion tensor was estimated using ordinary least squares applied to the preprocessed dMR images with low *b*-values (Procedure 1, *b* = 0, 700 s/mm^2^; Procedure 2, *b* = 0, 1500 s/mm^2^) to generate FA, MD maps for all subjects using the “*dtifit*” command in FSL software was adopted. The DTI and NODDI metrics maps were finally assessed to determine whether data were free from severe artifacts, such as gross geometric distortion, signal dropout, and bulk motion.

### Region of interest analysis

DTI and NODDI values were measured for a major bundle in WM according to the standard WM templates and atlas of Johns Hopkins University International Consortium for Brain Mapping (JHU ICBM-DTI-81) WM labels [38] (Supplementary Fig. 1). For localized regional WM, FA maps of all subjects were first realigned to the FA template of the JHU ICBM-DTI-81WM labels using the linear and nonlinear image registration tool in FSL software. In this way, the transformation parameters from the native FA to the FA template were obtained. The corresponding FA, MD, NDI, ISOVF, and ODI maps were subsequently realigned to the FA template space according to the transformation parameter obtained from registration between the native FA and FA template. Localized WM regions (genu, body, and splenium of the CC; superior cerebellar and cerebral peduncle; anterior and posterior limb of internal capsule; retrolenticular part of internal capsule; anterior, superior, and posterior corona radiata; posterior thalamic radiation; sagittal stratum; external capsule; cingulum cingulate gyrus and hippocampus; fornix stria terminalis; superior longitudinal fasciculus; superior fronto occipital fasciculus; uncinate fasciculus; and tapetum) were labeled according to JHU ICBM-DTI-81 WM labels. Lastly, each DTI and NODDI metrics was averaged over the region delineated by those atlases for all subjects.

### Harmonization methods

In this study, y(i,j,v)was the vth anatomical variable, in other words, diffusion metrics such as FA, MD, NDI, ISOVF, and ODI within the arbitrary WM label for scanner effect ifor jth subject. The harmonization method performed in this study was implemented using MATLAB (R2019a) and described.

ComBat harmonization: ComBat is an extension of GLM with an empirical Bayesian-based harmonization method that uses covariates regression for data harmonization. Although ComBat was originally used as a batch effect correction tool in genomics [27], this harmonization method has been introduced in neuroimaging recently, such as DTI data [28]. Fortine et al. harmonized the cortical thickness from multisite structural MRI [26] and FA and MD from multisite DTI [28] using ComBat to improve the statistical power. The ComBat formula can be described as:

(1) yi,j,v=av+XTi,j?v+?i,v+di,v?i,j,v,#1

where $a\left(v\right)$ is the average diffusion metric at the reference site within the vth anatomical variable, $?\left(v\right)$is the p?1vector of coefficients associated with the design matrix of biological covariates such as age and sex in this study, X(i,j)is the design matrix of the vth anatomical variable, and pis the number of biological covariates. ?(i,j,v)is the error term and follows a normal distribution with a mean of zero and a variance of $s^{2}(v)$. The terms $?\left(i,v\right)$and $d\left(i,v\right)$are the additive and multiplicative scanner effects on the vth anatomical variable. In ComBat harmonization, the terms ${?}^{*}\left(i,v\right)$and $d^{*}(i,v)$were estimated using an empirical Bayesian framework. The ComBat-harmonized values $y^{\mathrm{ComBat}}(i,j,v)$ can be described as:

(2) yComBati,j,v=yi,j,v-a^v-XTi,j?^v-?*i,vd*i,va^v+XTi,j?^v,#2

where $\overset{\text{\textasciicircum{}}}{?}\left(v\right)$and $\overset{\text{\textasciicircum{}}}{a}\left(v\right)$ represent estimated coefficients associated with the biological covariates and estimated population mean of the v th anatomical variable, respectively.

TS-GLM harmonization: GLM harmonization using TS data (TS-GLM) can differentiate sampling and measurement biases and eliminate measurement bias. While TS-GLM harmonization was originally proven to be useful for improving the SNR and harmonization of resting-state functional connectivity data [29], we followed this method for harmonizing dMRI metrics. The TS-GLM harmonization formula can be described as follows:

(3) yi,j,v=XSTi,j?sv+XPTi,j?Pv+?i,j,v#3

where ${?}_{P}\left(v\right)$is the participant factor and $X_{P}\left(i,j\right)$is the n?1vector of the participant indicator. ${?}_{s}\left(v\right)$represents the coefficient of the scanner effect factors such as site, model, and protocol factor that is, the measurement bias, and $X_{s}\left(i,j\right)$is the k?1vector of the scanner effect indicator. kwas the number of sites, models or protocols, and nwas the total number of traveling subjects. The inverse matrix for $X_{P}\left(i,j\right)$and $X_{s}\left(i,j\right)$were calculated to estimate the respective parameters. In this study, all the subjects were healthy and did not have an abnormality in the brain, so the sampling bias is likely reasonably small. Nevertheless, the design matrix of the GLM was rank-deficient. Thus, $\overset{\text{\textasciicircum{}}}{{?}_{s}}\left(v\right)$and $\overset{\text{\textasciicircum{}}}{{?}_{p}}\left(v\right)$were estimated using the Moore-Penrose pseudo-inverse matrix. The final TS-GLM harmonized values can be expressed as

(4) yTS-GLMi,j,v=yi,j,v-XSTi,j?s^v.#4

### Statistical analysis

Signal-to-noise ratio and contrast-to-noise ratio: Reportedly, the SNR and CNR of dMR images influences DTI and NODDI metrics [39]. Thus, the SNR in each regional WM was calculated for each of the scanner effects using preprocessed b=0s/mm^2^ images to evaluate the relation between SNR and scanner effects as follows:

(5) SNR=SIb=0SDb=0,#5

where ${\mathrm{SI}}_{b=0}$represents the mean signal intensity and ${\mathrm{SD}}_{b=0}$indicates the standard deviation in b=0(s/mm^2^) images. The CNR in each regional WM was calculated for each scanner effect using preprocessed b=2000s/mm^2^ images in the HARP protocol and preprocessed b=3000s/mm^2^ images in the CRHD protocol. The CNR for the dMRI was defined as follows:

(6) CNR=std(GP)std(res),#6

where $\mathrm{std}\left(\mathrm{GP}\right)$is the standard deviation of the Gaussian Process (GP) predictions and std(res)is the standard deviation of the residuals (the difference between the observations and the GP predictions).

A general mixed linear model (GLMM) was performed with SNR or CNR as dependent variables and model, protocol, and scan-rescan as independent variables to confirm the relation between SNR/CNR and the scanner effect. Hence, the site factor was removed to avoid instability due to multicollinearity in the model. Additionally, eta squared ( ${?}^{2}$), which is the proportion of variance ratio associated with each factor of an independent variable, was calculated.

Harmonization performance: To evaluate and compare the reproducibility of the different scanner effects before and after the harmonization methods, Cohen’s *d* effect size of the diffusion metric was calculated and the diffusion metric of the measurement bias and reproducibility for each different scanner effect including the site, model, protocol were evaluated. Cohen’s *d* formula can be described as follows:

(7) Sc=n1s12+n2s22n1+n2,#7

(8) Cohen's d=x¯1-x¯2Sc,#8

where $n_{1}$and $n_{2}$represent the number s of subjects in populations 1 and 2, respectively. ${\overset{\text{¯}}{x}}_{1}$and ${\overset{\text{¯}}{x}}_{2}$and $s_{1}$and $s_{2}$represent the average and standard deviation of each variable in populations 1 and 2, respectively. In this study, Cohen’s *d* for diffusion metrics was calculated between all site, model, and protocol combinations according to each scanner effect. Cohen’s *d* approaches a smaller value as the scanner effect decreases. If there is no scanner effect, Cohen’s *d* must equal 0. In this study, according to Cohen’s guideline [40], 0<d=0.2, 0.2<d=0.5, and 0.5<d=0.8were defined as small, medium, and large effects, respectively.

Retainability of biological information: To confirm the maintenance of biological information such as age and sex before and after the harmonization methods, Cohen’s *d* of diffusion metric between sex and the Spearman rank correlation coefficient $r_{s}$, between diffusion metric and sex were calculated for each different scanner effect, including the site, model, and protocol. Subsequently, the absolute difference between Cohen’s *d* and Spearman rank correlation coefficient $r_{s}$after Fisher’s Z -transformation before and after the harmonization methods were computed. In this study, according to Cohen’s guideline [40], $0.0<\left|?d\right|=0.2$, $0.2<\left|?d\right|=0.5$, and $0.5<\left|?d\right|=0.8$were defined as small, medium, and large effects, respectively. For comparing the correlation between before and after harmonization, Spearman rank correlation coefficient $r_{s}$was transformed to $\mathrm{Fishe}r^{'}\mathrm{s z}$. Additionally, to evaluate the variance of these relations between diffusion metrics and biological information in terms of the regression coefficient (i.e., beta coefficient), GLM based on least square fitting was performed as the dependent variable was diffusion metrics and the independent variable was age and sex as follows:

(9) Diffusion metric=Xage?age+Xsex?sex,#9

where Xis the design matric and ?is the regression coefficient for age and sex. Then, the absolute difference beta |??|before and after harmonization was calculated for age and sex. The retainability of biological information implies higher as |??|is smaller. Moreover, to compare the retainability of biological information of sex and age in brain WM between ComBat and TS-GLM harmonization, the absolute differences of the effect size, including Cohen’s *d* and $\mathrm{Fishe}r^{'}sz$, before and after harmonization were compared between ComBat and TS-GLM.

*Correlation with biological information:* To investigate the impact of harmonization on the correlation between diffusion metrics (i.e., DTI and NODDI metrics) and biological information (i.e., age, and sex), a GLM based on least square fitting was performed for data with different sites, models, and protocols. For the GLM, the dependent variable was diffusion metrics before or after harmonization and the independent variable was age and sex, according to formula (9). In line with previous reports on the correlation between diffusion metrics and biological information [41-43], there were significant correlations between diffusion metrics, including FA and NDI, and biological information in the cingulum cingulate gyrus (CGC), cingulum hippocampus (CGH), and fornix stria terminalis (Fx-ST). Therefore, this study targeted CGC, CGH, and Fx-ST and compared the beta coefficient of age and sex (i.e., ${?}_{\mathrm{age}}$and ${?}_{\mathrm{sex}}$) calculated by the GLM using each diffusion metrics before and after harmonization in these WM regions.

## RESULTS

### Signal-to-noise ratio and contrast-to-noise ratio

**Figure 1.**
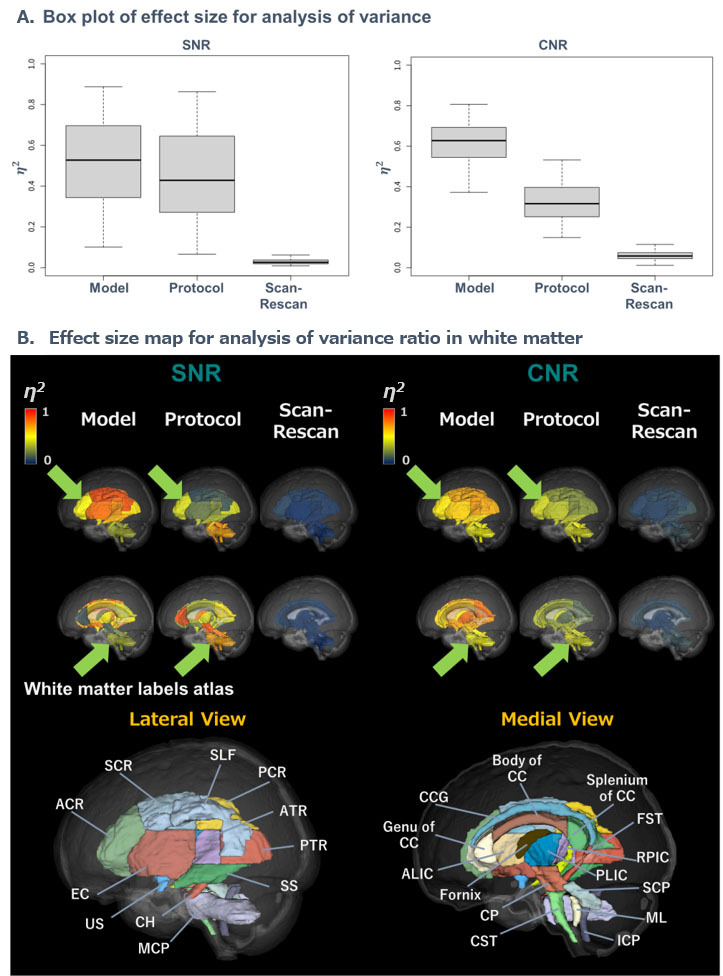
**Effect size of MRI measurements in Signal-to-noise ratio and contrast-to-noise ratio of diffusion-weighted magnetic resonance images. (A)** Box plots show the effect size of MRI measurements (MRI models, protocols and scan-rescan) in the analysis of variance ratio of SNR (left) and CNR (right). Y-axis represents the effect size, measured by eta squared () in SNR and CNR. The effect size is the largest in the effect of MRI models, followed by protocols and scan-rescan. **(B)** Shown are the effect size maps for MRI models, protocols, and scan-rescan in the analysis of variance in major WM regions of the standard WM template (JHU ICBM-DTI-81 WM labels). The effect size of MRI models and protocols, as represented by the eta squared () is relatively larger in the deeper part of WM, namely, CC, brainstem, and cerebrum peduncles (indicated by green arrows) as compared with those in more superficial WM regions like ACR, SCR and external capsules. Color bar shows the range of of each factor (MRI models, protocols, scan/rescan) in SNR and CNR values as the following: blue, *d* = 0; yellow *d* = 0.5; and red *d* = 1. Abbreviations: ACR, anterior corona radiata; CC; corpus callosum; CNR, contrast-to-noise ratio; SNR, signal-to-noise ratio; SCR, superior corona radiata; WM, white matter.

First, we have evaluated how various factors of the MRI (sites, models, and protocols) (Table 1) affect the basic quality of dMRI images, signal-to-noise ratio (SNR), and contrast-to-noise ratio (CNR) in the ICBM-DTI-81 WM labels atlas (Supplementary Fig. 1). Figure 1 shows MRI scanner effect onto the SNR and CNR in the WM regions of dMRI. Specifically, in Figure 1A, the largest effect size ( ${?}^{2}$) was found in models in both SNR and CNR, followed by protocols and scan-rescan. As shown in the effect size map for analysis of variance ratio ( ${?}^{2}$) in WM in Figure 1B, the effect size of eta squared of SNR, anterior corona radiata (ACR), superior corona radiata (SCR), posterior corona radiata (PCR), and external capsule (EC) were higher compared to corticospinal tract (CST), cerebral peduncle (CP), superior cerebellar peduncle (SCP), middle cerebellar peduncle (MCP), inferior cerebellar peduncle (ICP), and corpus callosum (CC). On the other hand, although the effect size of eta squared of CNR was higher in whole WM, the effect size of model factor is higher than that of protocol factor.

**Table 1:** Demographic characteristics of participants.

	All data	Scan-Rescan	Scanner effect		
			Between-Site	Between-Model	Between-Protocol
**Scan, n**	300	110 (55 / 55)	58	88	110
**Site, n**	12	12	3	2 (ATR or SWA)	1 (UTI or UTK)
**Scanner model, n**	7	1	1 (Prisma)	4	1 (Prisma)
**Protocol**	2	1	2 (HARP or CRHD)	1 (HARP)	2
**Participant**					
**N**	69	50	19	40	45
**Age (Mean ± SD), y**	31.6 ± 9.4	31.6 ± 10.0	31.7 ± 10.0	30.0 ± 8.2	32.6 ± 8.7
**Sex, M/F**	44/25	26/24	15/4	28/12	32/13
**Scan duration [Median (range)], d**	-	28 (0-176)	53 (0-117)	162 (0-221)	28 (0-168)

Abbreviations: SD, standard deviation

Supplementary Figure 2 shows SNR and CNR in the WM regions for each factor of site, scanner, protocol, and scan-rescan. For the site factor, SNR of UTI was lower than UTK and JTD in ACR, SCR, PCR, EC, SCP, CST, CP, MCP, ICP, and CC, while CNR of UTI was higher than that of UTK and JTD in those WM tracts. For the model factor, SNR and CNR of Prisma fit was lower than Skyra fit, Verio, and Verio dot in ACR, SCR, PCR, EC, SCP, MCP, ICP, and CC. For the protocol factor, SNR of HARP was higher than that of CRHD in CST, CP, MCP, ICP, and CC, while CNR of HARP was lower in ACR, SCR, PCR, EC, SCP, CST, CP, MCP, ICP, and CC. For the scan-rescan, there was almost difference in SNR and CNR.

### Evaluation of harmonization performance

**Figure 2.**
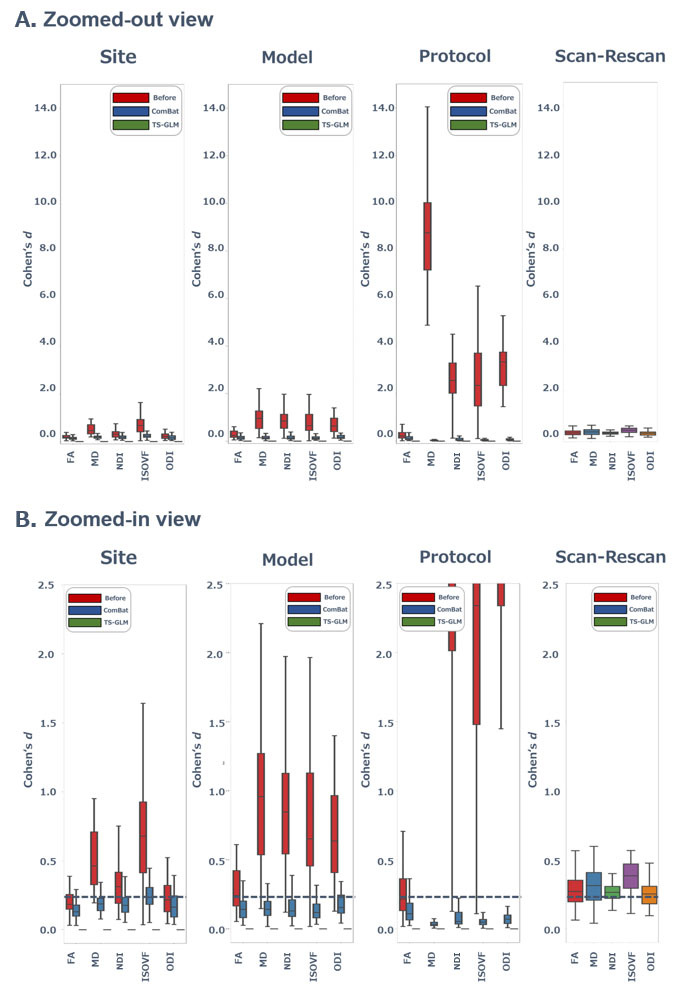
**Effect size of MRI measurements before and after the harmonization methods**. Box plots show the effect size (Cohen’s *d*) of the MRI measurements s (MRI sites, models, protocols and scan/rescan) in metrics of DTI and NODDI before (red) and after the harmonization (i.e., ComBat (blue) and TS-GLM (green)). The Cohen’s *d* is shown ranging from 0.0 to 15.0 in a zoom-out view **(A)**, and from 0.0 to 2.5 in zoom-in view **(B)**. Although the scanner effect of the between-protocol (*d* < 14.00) is larger than other effects (models (*d* < 3.00) and sites (*d* < 2.00)), ComBat also shows fairly well harmonization of dMRI metrics by decreasing protocol effect from *d* ≤ 14.00 to *d* < 0.20 while TS-GLM much harmonized them as seen small protocol effect (*d* < 0.01). Note that the effect size after TS-GLM harmonization is almost at the same level or less than those of scan-rescan data. Abbreviations: ComBat, combined association test; TS-GLM, traveling subject-general linear model; dMRI, diffusion-weighted magnetic resonance imaging; DTI, diffusion tensor imaging; GLM, general linear model; NODDI, neurite orientation dispersion and density imaging; TS, traveling subjects.

Second, we have evaluated the size of MRI measurement effects on DTI (fractional anisotropy (FA), mean diffusivity (MD)) and NODDI metrics (neurite density index (NDI), orientation dispersion index (ODI), isotropic volume fraction (ISOVF)). The effect size of scanner effect was evaluated by Cohen’s *d*. Figure 2 and Supplementary Table 1 show that the effect size (Cohen’s *d*) of scanner effect in each diffusion metrics in WM regions before and after the harmonization methods. When effect size of scanner effect was evaluated by Cohen’s *d*, the effect of protocols ( d<14.00) was the largest, followed by that of MRI models ( d<3.00) and sites ( d<2.00). Among multiple diffusion metrics, the effect size of model was smaller in FA ( d<1.00) than in other diffusion metrics. Compared to ComBat harmonization, TS-GLM largely decreased all scanner effect sizes for diffusion metric. Particularly, although effect of the protocol was the larger than other effects (sites and models), ComBat also achieved fairly well harmonization of dMRI metrics and decreased protocol effect from d=14.00to d<0.20while TS-GLM largely harmonized data and decreased the protocol effect to d<0.01. Furthermore, although ComBat well harmonized the model effect from d<3.00to d<0.50, TS-GLM remarkably harmonized one from d<3.00to d<0.01. Effect size after harmonization methods were almost of the same level or less than those of scan-rescan data (FA, median *d* = 0.21 (interquartile range, IQR: 0.12-0.31); MD, median *d* = 0.25 (IQR: 0.16-0.34); NDI, median *d* = 0.24 (IQR: 0.17-0.34); ISOVF) median *d* = 0.35 (IQR: 0.28-0.44); ODI, median *d* = 0.23 (IQR: 0.16-0.27)).

**Figure 3.**
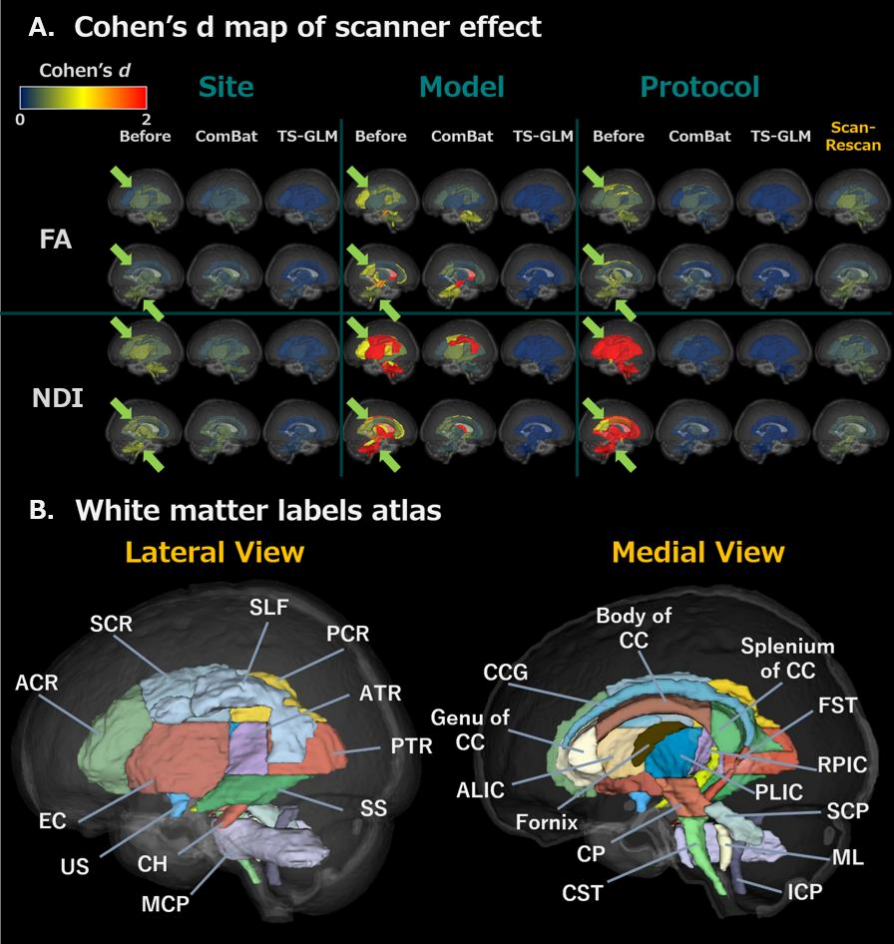
**Cohen’s *d* map of scanner effect in brain white matter before and after the harmonization methods**. Shown are the maps of scanner effects (Site, MRI models, Protocols) in the WM regions of the standard WM template (JHU ICBM-DTI-81) before and after the harmonization. Color shows effect size evaluated by Cohen’s *d* as the following: blue, *d* = 0; yellow, *d* = 1; and red, *d* = 2. The Cohen’s *d* of scanner effect before harmonization was relatively higher at green arrow. After harmonization using ComBat or TS-GLM, the Cohen’s *d* of scanner effect for both fractional anisotropy (FA) and intra-neurite volume fraction (NDI) decreased in whole WM up to that of scan-rescan in which Cohen’s *d* did not almost change between scan and rescan. However, TS-GLM more largely harmonized scanner effect up to Cohen’s *d* < 0.01 than ComBat. Abbreviations: ACR, anterior corona radiata; CC, corpus callosum; CP, cerebral peduncle; ComBat, combined association test; dMRI, diffusion-weighted magnetic resonance imaging; EC, external capsule; GLM, general linear model; MCP, middle cerebellar peduncle; PCR, posterior corona radiata; SCR, superior corona radiata; TS, traveling subjects; WM, white matter.

Figure 3 shows representative diffusion map such as FA and NDI of each scanner effect in brain WM before and after the harmonization methods. The Cohen’s *d* of scanner effect before harmonization was relatively higher in ACR, SCR, PCR, EC, SCP, CST, CP, MCP, ICP, and CC in the order of protocol, model, and site effect. Additionally, the Cohen’s *d* of NDI is higher than that of FA in whole WM before harmonization. After harmonization using ComBat or TS-GLM, the Cohen’s *d* of scanner effect decreased in whole WM up to that of scan-rescan in which Cohen’s d did not almost change between scan and rescan. These results were the same for the other diffusion metrics such as MD, ISOVF, and ODI (Supplementary Fig. 3). Thus, all diffusion metrics in WM tracts were harmonized via ComBat and TS-GLM. However, TS-GLM more largely harmonized scanner effect up to Cohen’s *d* < 0.01 than ComBat in especially ACR, SCR, PCR, EC, SCP, CST, CP, MCP, ICP, and CC.

### Retainability of biological information

**Figure 4.**
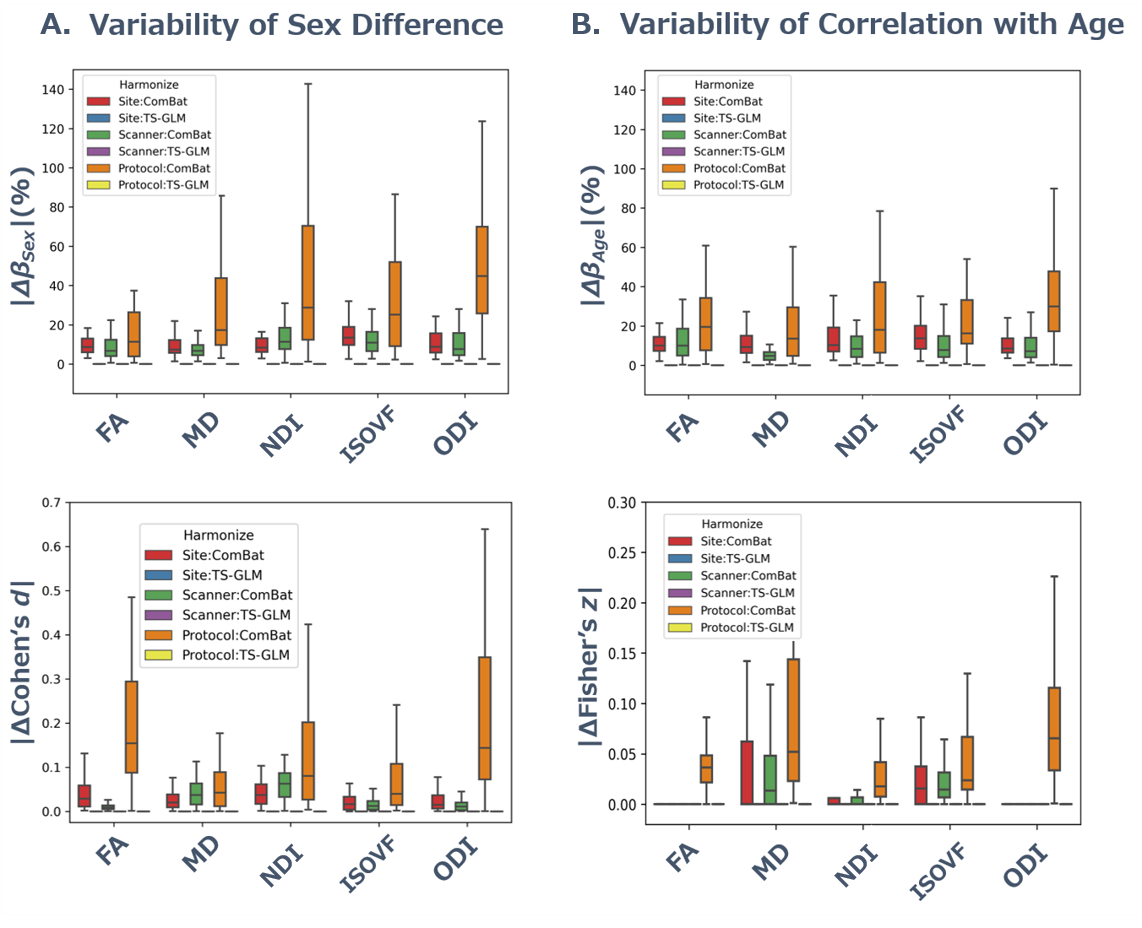
**Variability of biological information of sex and age in brain white matter before and after the harmonization methods**. Box plots show the variability of sex difference and correlation with age in all WM tracts for dMRI metrics before and after harmonization methods. **(A)** Y-axis represents the absolute difference of beta coefficient of sex calculated by GLM () and Cohen’s *d* () of sex difference before and after harmonization. **(B)** Y-axis represents the absolute difference of beta coefficient of age calculated by GLM () and Fisher’s *Z* of Spearman rank correlation coefficient () with age before and after harmonization. The absolute difference of Cohen’s *d* and of sex and age (i.e., the variability of biological information) before and after the harmonization methods was largest in the scanner effect of between-protocol, followed by between-model and between-site. Compared to ComBat, TS-GLM showed very small absolute differences in contributions and effects of sex and age, the retainability of biological information in TS-GLM harmonization was remarkably superior to that in ComBat. Abbreviations: dMRI, diffusion-weighted magnetic resonance imaging; ComBat, combined association test; GLM. General linear model; TS, traveling subjects; WM, white matter.

We estimated biological information retention before and after harmonization by estimating whether the absolute difference in contribution (beta coefficient calculated using GLM) and effect size (Fisher’s *Z* of Spearman’s $r_{s}$and Cohen’s *d*) of age or sex in WM metrics is reasonably small. Figure 4 and Supplementary Table 2 show the variability of biological effects of sex and age (i.e., the absolute difference of Cohen’s *d*, Fisher’s *Z*, and ?) in brain WM before and after the harmonization methods. The retainability of biological effects in brain WM gets better by TS-GLM than ComBat as seen in very small absolute differences in contributions and effects of sex and age. The variability of biological information of sex and age before and after the harmonization methods was largest in the scanner effect of between-protocol ( $\mathrm{median}\left|?d\right|<0.06,\mathrm{median}\left|?\mathrm{Fishe}r^{'}\mathrm{s z}\right|<0.15$), followed by between-model ( $\mathrm{median}\left|?d\right|<0.01,\mathrm{median}\left|?\mathrm{Fishe}r^{'}\mathrm{s z}\right|<0.06$) and between-site ( $\mathrm{median}\left|?d\right|<0.02,\mathrm{median}\left|?\mathrm{Fishe}r^{'}\mathrm{s z}\right|<0.02$). As Figure 4 and Supplementary Table 2 show, the retainability of biological information in TS-GLM harmonization was remarkably superior to that in ComBat. Moreover, the variance of these relations in terms of regression coefficient |??|showed a similar tendency to the above result in terms of Cohen’s *d* and correlation coefficients. Indeed, compared with ComBat, the retainability of biological information was significantly higher in TS-GLM for all diffusion metrics and scanner effects (*p* < 0.001, Supplementary Table 3). Hence, TS-GLM harmonization did not influence biological information at all.

### Correlation with biological information

We compared the beta coefficient of age and sex calculated using GLM before and after harmonization to investigate whether harmonization techniques can improve statistical power. Supplementary Table 4 and Supplementary Figure 4 show the correlation analysis results between diffusion metrics (i.e., FA and NDI) and biological information (i.e., sex and age) in brain WM before and after applying the harmonization methods. Despite harmonization, the beta coefficients were almost unchanged.

## DISCUSSION

To mitigate variability across multisite dMRI data and enable multisite study enlargement, this study evaluated the performance of ComBat and TS-GLM harmonization for DTI and NODDI metrics in WM. Consequently, the scanner effect of diffusion metrics was harmonized using ComBat and TS-GLM harmonization and reduced to the same level as that of scan-rescan. Although harmonization methods strongly mitigated the scanner effect, the biological effects of sex and age were shown to be highly retainable. Therefore, ComBat and TS-GLM show high harmonization performances for diffusion metrics in WM and retain biological information of age and sex.

As shown in Figure 3, although all diffusion metrics in WM regions were harmonized by ComBat and TS-GLM, the latter has stronger harmonization power than ComBat in ACR, SCR, PCR, EC, SCP, CST, CP, MCP, ICP, and CC. Although it is also reported that these WM regions have high CoVs of DTI and NODDI metrics in intermodels and protocols [20], TS-GLM could adequately harmonize the DTI and NODDI metrics in any major WM bundles with any scanner effect. As Figure 2 and Supplementary Table 1 show, the scanner effect of between-protocol was the largest, followed in order by model between-site. MD of DTI metrics was calculated using two procedures using different *b*-values. DTI reflects the Gaussian diffusion properties of water molecules in some biological tissue components, and the range of *b*-value reflects such Gaussian diffusion properties roughly from 500 to 1000 s/mm^2^. Over *b*-value of 1000 s/mm^2^ reflects non-Gaussian diffusion in some biological tissue components [44]. Thus, MD values can differ between procedures due to the large difference of *b*-values to compute DTI, and consequently, it might cause a large Cohen’s *d* of MD in between-protocol. In this study, despite the same protocol with the same *b*-shell, between-model differences in the scanning parameters are not negligible, for example, *b*-value component of diffusion time ( ?, ms), gradient pulse duration ( d, ms), echo time, bandwidth, echo spacing, and the number of gradient directions. These can influence not only the SNR and CNR in dMRI but also DTI and NODDI metrics [39, 45-47]. Indeed, the variances ( ${?}^{2}$) of SNR and CNR in model and protocol effects were much larger than that of scan-rescan (Fig. 1A). Consequently, between-model and -protocol had a larger Cohen’s *d* of diffusion metrics than between-site.

**Table 2:** Diffusion magnetic resonance imaging acquisition parameters.

Scanner model	Prisma	Prisma fit	Skyra	Skyra fit	Verio	Verio dot	Trio	Prisma	Prisma fit
**dMRI protocol**	Procedure 1							Procedure 2	
**Repetition time [ms]**	3600							3230	
**Echo time [ms]**	79.0		89.0				94.0	89.2	
**Flip angle [deg]**	90							78	
**Field of view [mm]**	204 × 204 × 144							210 × 210 × 138	
**Matrix size**	120 × 120							140 × 140	
**No. of slices (axial)**	84							92	
**Voxel size [mm]**	1.7 × 1.7 × 1.7							1.5 × 1.5 × 1.5	
**Partial Fourier**	6/8							0.75	
**Multiband factor**	3							4	
**Bandwidth [Hz/Px]**	1984	1544		1436		1736		1700	
**Echo spacing [msec]**	0.62	0.74		0.78		0.70		0.69	
**b-values [s/mm^2^]**	0/700/2000								0/1500/3000
**No. of directions (AP)**	5/16/32	7/20/40							7/47/46
**Diffusion time (Δ) [ms]**	39.21		43.61		43.44	43.52	46.36	43.38	
**Gradient pulse duration (δ) [ms]**	12.91		26.88	26.29	26.31	30.03		13.7	
**No. of directions (PA)**	6/16/32		8/20/40					7/47/46	
**Scan time [min:sec]**	7:02		9:44					11:18	

Abbreviations: dMRI, diffusion-weighted magnetic resonance imaging

Despite scan-rescan, it is reported that the intrasubject CoVs in WM were around 0.5% in DTI metrics [24, 48] and around 1% in CoV of NODDI metrics in major WM tracts [20, 23, 49]. As with previous reports, although the scan-rescan data in this study excluded the scanner effect, that is, not only sampling bias but also measurement bias, Cohen’s *d* was not 0, contrary to expectations. It implies that there could be additional factors to be present that could have affected the reproducibility such as image analysis error, individual errors, and measurement bias. However, as shown in Figure 2 and Supplementary Table 1, the TS-GLM harmonization method indicated a lower Cohen’s *d* of the scanner effect than that of scan-rescan. This can be because TS-GLM can harmonize those additional factors that can occur in scan-rescan without modeling. Conversely, although ComBat harmonization also tended to be higher reproducibility than the scan-rescan case, the harmonization performance of TS-GLM was superior to that of ComBat.

As shown in Figure 4 and Supplementary Table 2, the variability of biological information of sex (Cohen’s *d*) and age ( $?\mathrm{Fishe}r^{'}\mathrm{s z}$of correlation coefficient *r_s_*) in brain WM was kept small and maintained well after harmonization. Moreover, the variance of this relation in terms of regression coefficient |??|showed a similar tendency to the above result in terms of Cohen’s *d* and correlation coefficient. Hence, as reported in the structural MRI study targeting cortical and subcortical thickness and volume [30] and functional MRI study targeting functional connectivity [29], in our dMRI study targeting DTI and NODDI metrics, TS-GLM harmonization did not influence biological information since TS-GLM could separate sampling and measurement bias of the scanner effect and harmonize only measurement bias. Nevertheless, although it is difficult for ComBat to estimate individual effects and separate the measurement bias and individual effects, the harmonization performance was also sufficient in this study.

Such variabilities in diffusion metrics should be noted and considered because they can resemble subtle pathological changes in brain disorders. For instance, FA, which is a representative measure of DTI, shows changes compared with healthy controls of the same order as intramodel changes in the WM in schizophrenia [14] and bipolar disorder [13]. Additionally, multisite NODDI metrics have model variabilities in the same order of subtle pathological changes as in mild traumatic brain injury (mTBI) [50]. Thus, it is crucial to develop a technique to reduce the variability across multisite dMRI data to evaluate these subtle pathological changes accurately.

For multisite studies targeting disease, harmonization must reduce the measurement bias to detect pathological changes. In this study, we confirmed whether ComBat and TS-GLM could harmonize the scanner effect to reach the level that can detect the pathological changes. Regarding the psychiatric disorder that is known for the subtle pathological change, Kelly et al. [14] reported that compared with HC, FA of WM in schizophrenia was lower (*d* = -0.42). For bipolar disorder, FA of WM was lower (*d* = -0.43 to -0.54) [13]. Moreover, besides the psychiatric disorder, Palacios et al. [50] investigated the pathological change of DTI and NODDI metrics between mTBI and HC in WM. mTBI population showed that FA and NDI were lower (FA, *d* = -0.96 to -1.99; NDI, *d* = -0.31 to -1.24) and MD and ISOVF were higher (MD, *d* = 0.93–3.98; ISOVF, *d* = 0.94–2.05) than in HC population. Conversely, although our study showed that the scanner effect was larger than the above pathological changes in between-site ( d<2.00), between-model ( d<3.00), and between-protocol ( d<14.00), ComBat and TS-GLM harmonized these scanner effect up to the level that can detect the pathological change (Fig. 4 and Supplementary Table 1). Furthermore, the effect size of sex differences was maintained even after harmonization ( $\left|?d\right|<0.15$, Fig. 4 and Supplementary Table 2). These results indicate that ComBat and TS-GLM could reasonably harmonize the scanner effect but also maintain biological change.

In our study, we demonstrated that ComBat and TS-GLM maintained the biological information of age as well as sex after harmonization ( $\left|?\mathrm{Fishe}r^{'}\mathrm{s z}\right|<0.05$, Fig. 4 and Supplementary Table 2). Moreover, compared with ComBat, the retainability of biological information was significantly higher in TS-GLM for all diffusion metrics and scanner effects (*p* < 0.001, Supplementary Table 3). This high retainability of biological information is an important feature of harmonization, which may be useful to increase sensitivity to pathological characteristics in multisite studies. Indeed, the current study with ComBat and TS-GLM harmonization showed the variability of biological effects of sex and age (i.e., the absolute difference of Cohen’s *d*, Fisher’s *Z*, and ?) in brain WM before and after the harmonization methods up to be able to detect smaller age effect in diffusion metrics ( $\mathrm{median}\left|?d\right|<0.06,\mathrm{median}\left|?\mathrm{Fishe}r^{'}\mathrm{s z}\right|<0.15$). For instance, Andica et al. [51] reported that the correlation in ASD between NDI in WM and Autism Spectrum Quotient score was negative ( r=-0.40to-0.41). Moreover, Lang et al. [52] showed that the correlation in schizophrenia between FA of WM and Positive and Negative Syndrome Scale was negative ( r=-0.38to-0.50). Furthermore, the work of Saxena et al targeting BD [53] showed that the correlation between FA of WM and Life History of Aggression was negative ( r=-0.42). Thus, ComBat and TS-GLM have the feasibility to promote multisite study using dMRI and could make it successful. A future study will confirm the retainability of the above correlation as well as pathological changes in diffusion metrics and clinical scores using some patient data.

ComBat has several challenges for DTI and NODDI harmonization. First, although Fortin et al. [28] suggested ComBat harmonization of DTI for the first time, their tried to harmonize FA and MD and showed that ComBat worked well for DTI harmonization. Our study showed that ComBat can effectively harmonize NODDI as well as DTI. Second, although DTI metrics were harmonized voxel-wise after registering individuals to the template brain, it is possible that the DTI metrics in each voxel were not correctly harmonized due to misregistration among subjects’ brains [28]. A previous study reported difficulty with voxel-based harmonization, as misregistration of over 1.0 mm (≥1 voxel) is not acceptable for correct harmonization. Our study applied harmonization to region of interest-wise diffusion metrics to mitigate misregistration. Third, the prior study used DTI data acquired as part of two large, independent imaging studies with images acquired on different models with different imaging protocols [28]. The DTI metrics were harmonized using ComBat between populations matched across studies for age, gender, ethnicity, and handedness. Nevertheless, the datasets included measurement bias as well as sampling bias. Thus, it remains unclear whether ComBat could remove only measurement bias, thereby correctly retaining the individual effect. Our study showed that ComBat using TS data can reasonably remove only measurement bias. Fourth, their study using ComBat did not confirm the retainability of biological information [28]. Additionally, this study showed that ComBat and TS-GLM can not only harmonize the scanner effect but also maintain biological information. Finally, previous studies on harmonization, including the work by Fortin et al., did not separate the scanner effect into between-site, -model, and -protocol effects and evaluate them separately [28]. Our study confirmed that ComBat and TS-GLM are useful for harmonizing the between-site, -model, and -protocol scanner effect.

Herein, the harmonization power of TS-GLM based on TS data model was more powerful than that of ComBat. Moreover, in the work by Yamashita, et al. [29] that developed TS-GLM harmonization and applied it to resting-state functional connectivity data, TS-GLM outperformed ComBat in harmonization of multisite rs-fMRI data. TS-GLM harmonization achieved reduction in measurement bias by 29%, compared with 3% in the second highest value for the ComBat method. Thus, TS-GLM may be superior for properly estimating measurement bias and harmonizing multisite dMRI data (i.e., DTI, NODDI metrics), as well as rs-fMRI data (i.e., functional connectivity data), for the development of a wide range of final applications.

There are some limitations in this study. First, this study did not use the independent TS validation dataset for external validation, including datasets with a diverse range of ethnicities and demographics and patients with some pathology for harmonization to confirm the general performance of harmonization. However, it was extremely challenging to use patients as traveling subjects due to ethical considerations. Nevertheless, at present, there is no study including an external TS validation dataset [26, 28, 30, 54-56]. Moreover, the number of TS in our study was much higher than that in previous studies (n < 20) [29, 30, 54, 55]. TS-GLM could lead to overfitting and might not be robust enough for different populations. Furthermore, as our study targeted 7 Siemens models of a single 3T MRI vendor (i.e., Siemens), our findings might not generalize to all MRI vendors, models, and protocols. These concerns should be verified using an external validation dataset. Second, although TS-GLM harmonization can mitigate the measurement bias at the time of scanning, the TS-scanning intervals might lead to sampling bias caused by structural brain changes with normal aging. This study targeted healthy Japanese participants with age range from 20 to 60 years and had a maximum scan interval of 259 days, changing 1% annually [57]. Therefore, the effect of scan interval in this study was presumed to be negligible. Third, at present, although no golden standard or ground truth is available for the preprocessing of DTI and NODDI, there are several preprocessing steps. However, differences in image preprocessing affects reproducibility [58]. Fourth, although we compared the beta coefficient of age and sex calculated using GLM in the correlation analysis between diffusion metrics (i.e., FA and NDI) and biological information (i.e., sex and age) in brain WM before and after harmonization to investigate whether these techniques improve statistical power, all beta coefficients were almost unchanged. Although this study evaluated harmonization performance for diffusion metrics using cross-sectional TS data, future studies should use longitudinal TS data to precisely investigate whether harmonization techniques are useful for improving statistical power. Fifth, the optimal sample size is unknown for ComBat and TS-GLM. However, determining the optimal sample size is difficult because the sample size depends on the dataset. As the distance (similarity) is closer among the scanner effects, a large sample size might be required to exactly identify the measurement bias and eliminate it from dMRI metrics. Moreover, the optimal sample size has to be determined considering dataset with a diverse range of ethnicities, demographics, and patients with some pathology for ensuring the general performance of harmonization. Finally, although TS-GLM needs TS subjects, major databases do not include TS subject data, such as Alzheimer’s Disease Neuroimaging Initiative (https://adni.loni.usc.edu/), Parkinson’s Progression Markers Initiative www.ppmi-info.org/), and The Lifespan HCP in Aging (www.humanconnectome.org/study/hcp-lifespan-aging). Therefore, TS-GLM cannot be applied to these databases. In this study, ComBat also showed promising and feasible harmonization for dMRI such as DTI and NODDI and can be useful for the above already existing database.

In conclusion, our study showed that while ComBat and TS-GLM harmonization methods are useful for mitigating the scanner effect of site, model, and protocol for DTI and NODDI metrics in WM, either of harmonization methods had high retainability of biological effect. Thus, we propose that a multisite study targeting large cohorts can correctly detect subtle pathological changes after harmonizing DTI and NODDI metrics by maintaining pathological effects.

## Supplementary Materials

www.aginganddisease.org/EN/10.14336/AD.2023.1020. TS data are available as a part of the Brain/MINDS Beyond human brain MRI project (https://hbm.brainminds-beyond.jp).
