# Supplementary material for: Traveling Subject-Informed Harmonization Increases Reliability of Brain Diffusion Tensor and Neurite Mapping
Source: Aging Dis. 2023 Nov 15;15(6):2770–85. doi: 10.14336/AD.2023.1020 (PMC11567268; doi:10.14336/AD.2023.1020)
Supplement: Supplementary file 1 — www.aginganddisease.org/EN/10.14336/AD.2023.1020. TS data are available as a part of the Brain/MINDS Beyond human brain MRI project (https://hbm.brainminds-beyond.jp). [file AD-15-6-2770-s.pdf]

SUPPLEMENTARY DATA

Traveling Subject-Informed Harmonization Increases Reliability of Brain Diffusion Tensor and Neurite Mapping

Yuya Saito, Koji Kamagata, Christina Andica, Norihide Maikusa, Wataru Uchida, Kaito Takabayashi, Seina Yoshida, Akifumi Hagiwara, Shohei Fujita, Toshiaki Akashi, Akihiko Wada, Ryusuke Irie, Keigo Shimoji, Masaaki Hori, Kouhei Kamiya, Shinsuke Koike, Takuya Hayashi, Shigeki Aoki

© 2023. Saito Y et al. Published online at http://www.aginganddisease.org/EN/10.14336/AD.2023.1020

SUPPLEMENTARY DATA

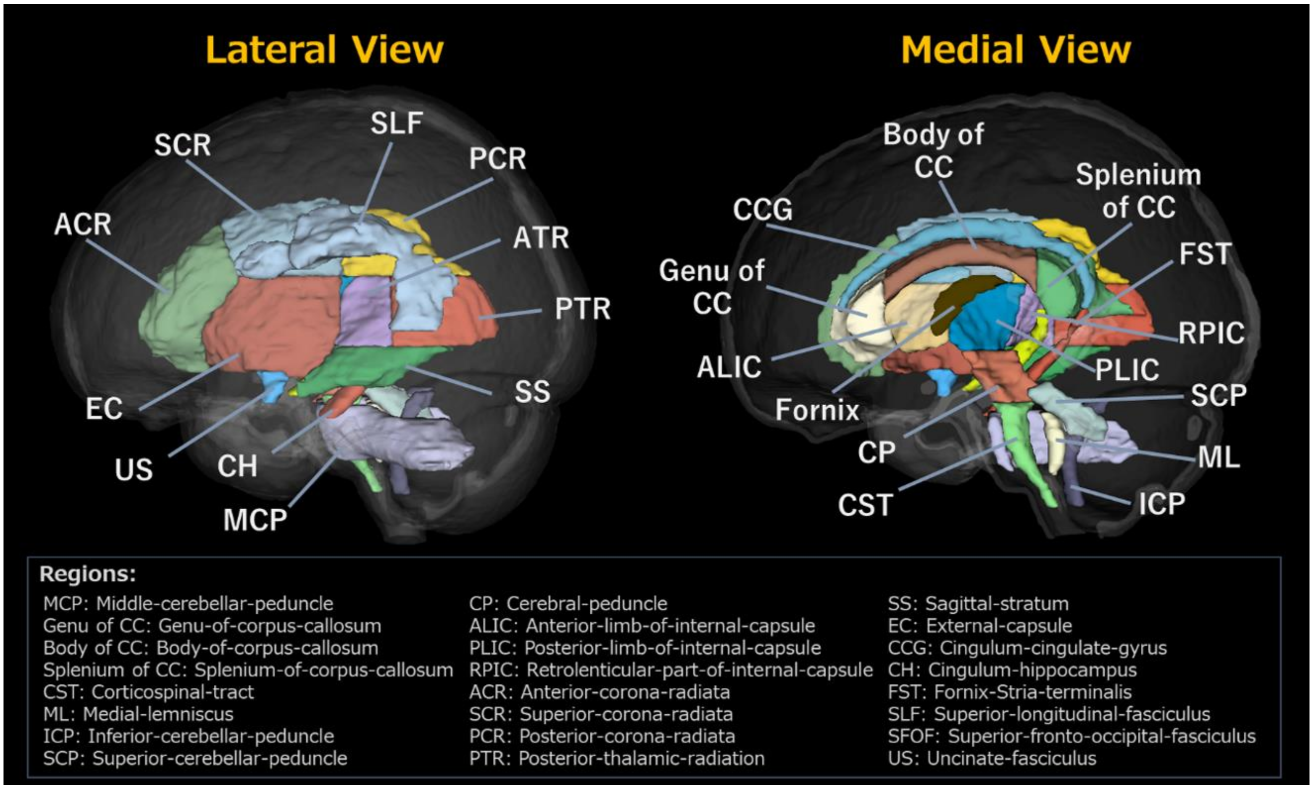

Supplementary Figure 1. ICBM-DTI-81 atlas of white matter (WM) labels WM tract regions of interest based on the ICBM-DTI-81 white matter label atlas for cerebral regions.

© 2023. Saito Y et al. Published online at http://www.aginganddisease.org/EN/10.14336/AD.2023.1020

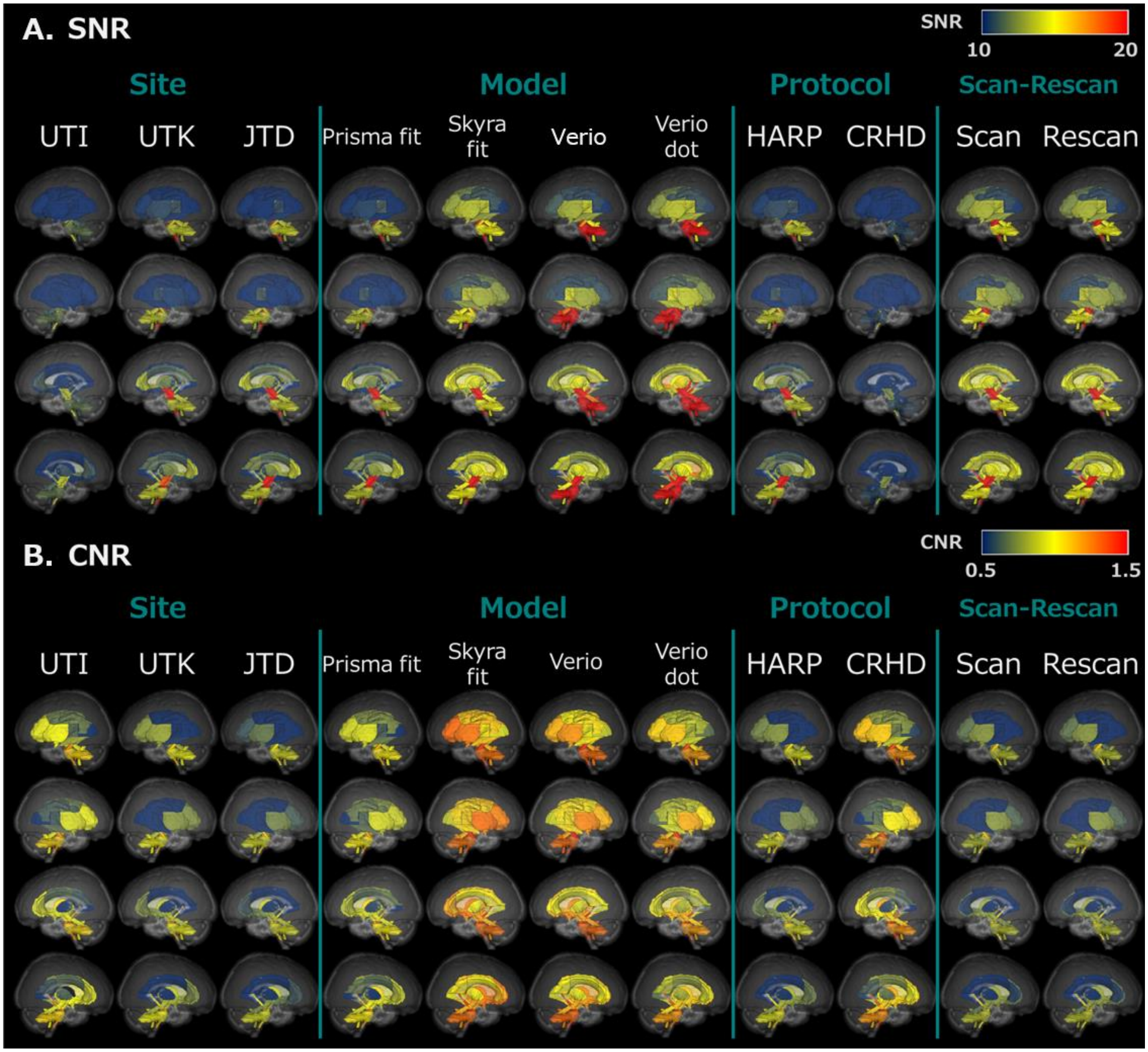

Supplementary Figure 2. Signal-to-noise ratio and contrast-to-noise ratio maps of scanner effects for brain white matter in diffusion-weighted magnetic resonance images. The upper and lower rows show lateral and medial images, respectively. The columns indicate the site, model, protocol, and scan–rescan results. The colors indicate the signal-to-noise ratio (SNR) and contrast-to-noise ratio (CNR) values as follows: red, SNR = 10, CNR = 0.5; yellow, SNR = 15, CNR = 1.0; and blue, SNR = 20, CNR = 1.5. For the site factor, the SNR of UTI was lower than that of UTK and JTD in ACR, SCR, PCR, EC, SCP, CST, CP, MCP, ICP, and CC, whereas the CNR of UTI was higher than that of UTK and JTD in ACR, SCR, PCR, EC, SCP, CST, CP, MCP, ICP, and CC. For the model factor, the SNR and CNR of the Prisma fit were lower than the Skyra fit, Verio, and Verio dot in ACR, SCR, PCR, EC, SCP, MCP, ICP, and CC. For the protocol factor, the SNR of HARP was higher than that of CRHD in CST, CP, MCP, ICP, and CC, while the CNR of HARP was lower in ACR, SCR, PCR, EC, SCP, CST, CP, MCP, ICP, and CC. For the scan–rescan, there was almost no difference in SNR and CNR. Abbreviations: UTI, University of Tokyo IRCN; UTK, University of Tokyo ECS (Komaba Campus); JTD; Juntendo Hospital, HARP, Beyond Human Brain MRI project harmonized protocol; CRHD; Human Connectome Project protocol for Connectomes Related to Human Disease.

SUPPLEMENTARY DATA

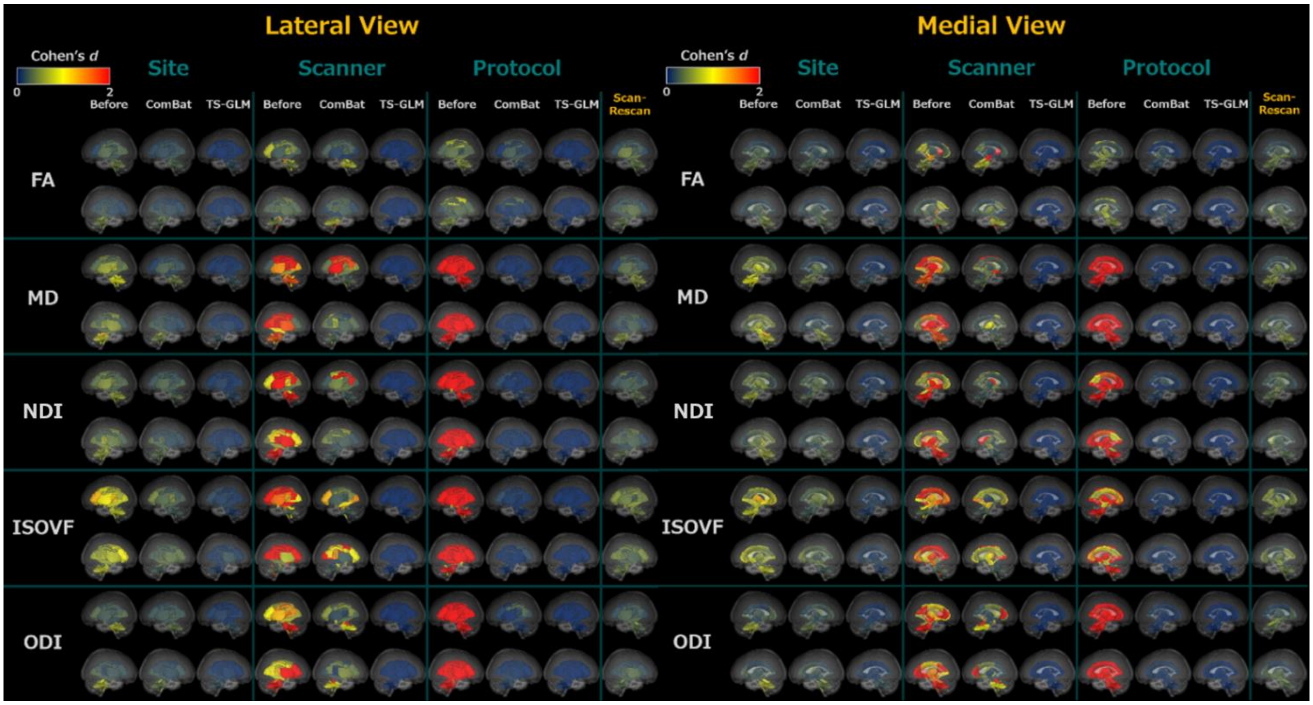

Supplementary Figure 3. Cohen's *d* map of the scanner effect in brain white matter before and after the harmonization methods. The left and right blocks show lateral and medial images, respectively. The columns indicate the site, model, and protocol. The rows indicate diffusion tensor imaging, neurite orientation dispersion, and density imaging metrics. The colors indicate Cohen's *d* values as follows: blue, $d = 0$; yellow, $d = 1$; and red, $d = 2$. Among all white matters, the green arrow shows a white matter region with relatively higher Cohen's *d* than other regions. Cohen's *d* of the scanner effect before harmonization was relatively higher in ACR, SCR, PCR, EC, SCP, CST, CP, MCP, ICP, and CC in the order of protocol, model, and site effect. In addition, Cohen's *d* to NDI was higher than that of FA in the overall WM before harmonization. After harmonization using ComBat or TS-GLM, the Cohen's *d* of the scanner effect decreased in the overall WM to that of scan–rescan; Cohen's *d* showed almost no change between scan and rescan. These results were the same for the other diffusion metrics, such as MD, ISOVF, and ODI. Abbreviations: ComBat, combined association test; TS-GLM, traveling subject-general linear model; FA, fractional anisotropy; MD, mean diffusivity; NDI, neurite density index; ISOVF, isotropic volume fraction; ODI, orientation dispersion index; ACR, anterior corona radiata; SCR, superior corona radiata; PCR, posterior corona radiata; EC external capsule; CST, corticospinal tract; CP, cerebral peduncle; SCP, superior cerebellar peduncle; MCP, middle cerebellar peduncle (MCP); ICP, inferior cerebellar peduncle; CC, corpus callosum (CC).

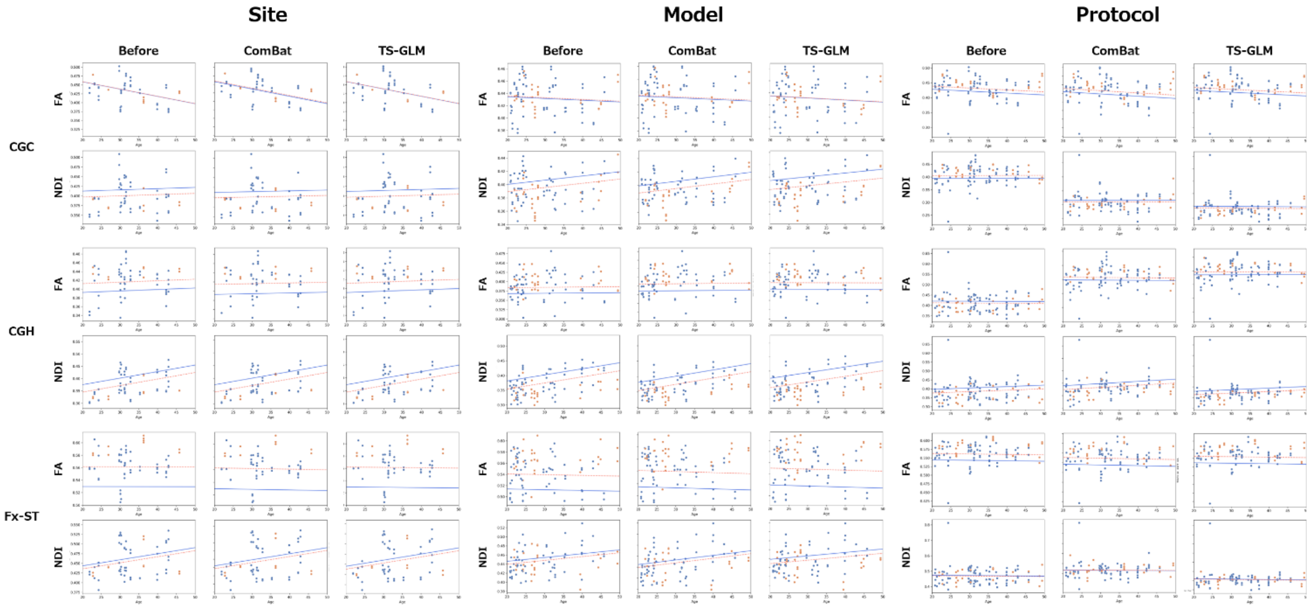

© 2023. Saito Y et al. Published online at http://www.aginganddisease.org/EN/10.14336/AD.2023.1020

SUPPLEMENTARY DATA

Supplementary Figure 4. Scatter plot of the diffusion metrics and biological information of sex and age in brain WM before and after the harmonization methods. The y-axis indicates the diffusion metrics, while the x-axis indicates age. The blue and red lines were estimated using linear regression for males and females, respectively. All beta coefficients were not almost changed despite harmonization. Abbreviations: ComBat, combined association test; TS-GLM, traveling subject-general linear model; FA, fractional anisotropy; MD, mean diffusivity; NDI, neurite density index; ISOVF, isotropic volume fraction; ODI, orientation dispersion index; CGC, cingulum cingulate gyrus; CGH, cingulum hippocampus; Fx-ST, fornix stria terminalis.

Supplementary Table 1. Cohen's *d* values of the site effect for each diffusion metrics before and after the harmonization methods
(a) Scanner effect of site, model, and protocol differences.

Scanner effect	Map	Before [Median (IQR)]	ComBat [Median (IQR)]	TS-GLM [Median (IQR)]
Between -Site	FA	0.18 (0.15–0.26)	0.13 (0.10–0.18)	0.00 (0.00–0.00)
	MD	0.46 (0.33–0.71)	0.19 (0.14–0.23)	0.00 (0.00–0.00)
	NDI	0.31 (0.19–0.42)	0.18 (0.13–0.23)	0.00 (0.00–0.00)
	ISOVF	0.68 (0.41–0.93)	0.23 (0.18–0.31)	0.00 (0.00–0.00)
	ODI	0.22 (0.13–0.32)	0.16 (0.09–0.25)	0.00 (0.00–0.00)
Between -Mode	FA	0.24 (0.17–0.42)	0.14 (0.08–0.20)	0.00 (0.00–0.00)
	MD	0.96 (0.54–1.27)	0.14 (0.10–0.20)	0.00 (0.00–0.00)
	NDI	0.85 (0.54–1.13)	0.13 (0.09–0.21)	0.00 (0.00–0.00)
	ISOVF	0.65 (0.46–1.13)	0.12 (0.08–0.18)	0.00 (0.00–0.00)
	ODI	0.64 (0.41–0.96)	0.15 (0.11–0.24)	0.00 (0.00–0.00)
Between -Protocol	FA	0.22 (0.13–0.36)	0.11 (0.06–0.19)	0.00 (0.00–0.00)
	MD	8.75 (7.19–10.02)	0.03 (0.02–0.05)	0.00 (0.00–0.00)
	NDI	2.55 (2.01–3.28)	0.05 (0.04–0.12)	0.00 (0.00–0.00)
	ISOVF	2.34 (1.48–3.70)	0.05 (0.03–0.07)	0.00 (0.00–0.00)
	ODI	3.33 (2.34–3.74)	0.07 (0.04–0.10)	0.00 (0.00–0.00)

(b) Scan–rescan

Map	Scan–rescan [Median (IQR)]
FA	0.21 (0.12–0.31)
MD	0.25 (0.16–0.34)
NDI	0.24 (0.17–0.34)
ISOVF	0.35 (0.28–0.44)
ODI	0.23 (0.16–0.27)

Abbreviations: ComBat, combined association test; TS-GLM, traveling subject-general linear model; IQR, interquartile range; FA, fractional anisotropy; MD, mean diffusivity; NDI, neurite density index; ISOVF, isotropic volume fraction; ODI, orientation dispersion index.

© 2023. Saito Y et al. Published online at http://www.aginganddisease.org/EN/10.14336/AD.2023.1020

SUPPLEMENTARY DATA

Supplementary Table 2. Variability values of biological information of sex and age in brain white matter before and after the harmonization methods.

		|ΔCohen's d| [Median (IQR)]		|$\Delta Fisher's\ z$| [Median (IQR)]	
**Scanner effect**	**Map**	**ComBat**	**TS-GLM**	**ComBat**	**TS-GLM**
**Between -Site**	**FA**	0.00 (0.00–0.00)	0.00 (0.00–0.00)	0.00 (0.00–0.01)	0.00 (0.00–0.00)
	**MD**	0.02 (0.01–0.02)	0.00 (0.00–0.00)	0.02 (0.01–0.03)	0.00 (0.00–0.00)
	**NDI**	0.00 (0.00–0.02)	0.00 (0.00–0.00)	0.01 (0.01–0.02)	0.00 (0.00–0.00)
	**ISOVF**	0.00 (0.00–0.02)	0.00 (0.00–0.00)	0.02 (0.01–0.03)	0.00 (0.00–0.00)
	**ODI**	0.01 (0.00–0.02)	0.00 (0.00–0.00)	0.01 (0.01–0.03)	0.00 (0.00–0.00)
**Between -Mode**	**FA**	0.00 (0.00–0.00)	0.00 (0.00–0.00)	0.01 (0.00–0.01)	0.00 (0.00–0.00)
	**MD**	0.01 (0.00–0.02)	0.00 (0.00–0.00)	0.04 (0.02–0.06)	0.00 (0.00–0.00)
	**NDI**	0.01 (0.00–0.04)	0.00 (0.00–0.00)	0.06 (0.03–0.09)	0.00 (0.00–0.00)
	**ISOVF**	0.00 (0.00–0.01)	0.00 (0.00–0.00)	0.01 (0.00–0.02)	0.00 (0.00–0.00)
	**ODI**	0.00 (0.00–0.00)	0.00 (0.00–0.00)	0.01 (0.00–0.02)	0.00 (0.00–0.00)
**Between -Protocol**	**FA**	0.04 (0.02–0.05)	0.00 (0.00–0.00)	0.15 (0.09–0.29)	0.00 (0.00–0.00)
	**MD**	0.02 (0.01–0.07)	0.00 (0.00–0.00)	0.04 (0.01–0.09)	0.00 (0.00–0.00)
	**NDI**	0.05 (0.02–0.14)	0.00 (0.00–0.00)	0.08 (0.03–0.20)	0.00 (0.00–0.00)
	**ISOVF**	0.02 (0.01–0.04)	0.00 (0.00–0.00)	0.04 (0.01–0.11)	0.00 (0.00–0.00)
	**ODI**	0.06 (0.03–0.10)	0.00 (0.00–0.00)	0.14 (0.07–0.35)	0.00 (0.00–0.00)

Abbreviations: ComBat, combined association test; TS-GLM, traveling subject-general linear model; IQR, interquartile range; FA, fractional anisotropy; MD, mean diffusivity; NDI, neurite density index; ISOVF, isotropic volume fraction; ODI, orientation dispersion index.

Supplementary Table 3. Comparison of the retainability of biological information of sex and age in brain white matter between the ComBat and TS-GLM harmonization methods.

		**Age**			**Sex**		
		**Site**	**Model**	**Protocol**	**Site**	**Model**	**Protocol**
**FA**	***Z***	−2.21	−2.53	−5.97	−5.97	−6.03	−6.03
	P	<0.05	<0.05	<0.001	<0.001	<0.001	<0.001
**MD**	***Z***	−5.78	−5.65	−6.03	−5.84	−6.03	−6.03
	P	<0.001	<0.001	<0.001	<0.001	<0.001	<0.001
**NDI**	***Z***	−4.20	−4.55	−6.03	−5.51	−6.03	−6.03
	P	<0.001	<0.001	<0.001	<0.001	<0.001	<0.001
**ISOVF**	***Z***	−4.37	−3.19	−5.84	−5.58	−6.03	−6.03
	P	<0.001	<0.005	<0.001	<0.001	<0.001	<0.001
**ODI**	***Z***	−4.86	−2.68	−6.03	−5.78	−6.03	−6.03
	P	<0.001	<0.01	<0.001	<0.001	<0.001	<0.001

Abbreviations: FA, fractional anisotropy; MD, mean diffusivity; NDI, neurite density index; ISOVF, isotropic volume fraction; ODI, orientation dispersion index.

© 2023. Saito Y et al. Published online at http://www.aginganddisease.org/EN/10.14336/AD.2023.1020

SUPPLEMENTARY DATA

Supplementary Table 4. Correlation analysis between diffusion metrics and biological information of sex and age in brain white matter before and after the harmonization methods

(a) Age

			Site			Model			Protocol		
			**β**	***t*-value**	***p*-value**	**β**	***t*-value**	***p*-value**	**β**	***t*-value**	***p*-value**
**FA**	**CGC**	**Before**	−0.002	−3.091	0.003	0.000	−0.796	0.428	−0.001	−1.636	0.105
		**ComBat**	−0.002	−3.401	0.001	0.000	−0.745	0.459	−0.001	−1.759	0.081
		**TS-GLM**	−0.002	−3.120	0.003	0.000	−0.948	0.346	−0.001	−1.637	0.105
	**CGH**	**Before**	0.000	0.518	0.606	0.000	0.205	0.838	0.000	0.056	0.955
		**ComBat**	0.000	0.264	0.793	0.000	0.256	0.799	0.000	−0.189	0.850
		**TS-GLM**	0.000	0.463	0.645	0.000	−0.074	0.942	0.000	0.064	0.949
	**Fx-ST**	**Before**	0.000	−0.019	0.985	0.000	−0.344	0.731	0.000	−0.443	0.659
		**ComBat**	0.000	−0.243	0.809	0.000	−0.535	0.594	0.000	−0.633	0.528
		**TS-GLM**	0.000	−0.143	0.887	0.000	−0.488	0.627	0.000	−0.477	0.634
**NDI**	**CGC**	**Before**	0.000	0.420	0.676	0.001	2.096	0.039	0.000	−0.145	0.885
		**ComBat**	0.000	0.280	0.780	0.001	2.665	0.009	0.000	0.047	0.963
		**TS-GLM**	0.000	0.336	0.738	0.001	2.153	0.034	0.000	−0.224	0.823
	**CGH**	**Before**	0.003	3.080	0.003	0.002	4.683	0.000	0.001	1.343	0.182
		**ComBat**	0.003	3.009	0.004	0.002	5.698	0.000	0.001	2.406	0.018
		**TS-GLM**	0.003	3.209	0.002	0.002	4.721	0.000	0.001	1.963	0.052
	**Fx-ST**	**Before**	0.002	1.957	0.055	0.001	2.232	0.028	0.000	−0.326	0.745
		**ComBat**	0.001	1.830	0.073	0.001	2.540	0.013	0.000	−0.204	0.839
		**TS-GLM**	0.002	1.903	0.062	0.001	2.108	0.038	0.000	−0.505	0.615

(b) Sex

			Site			Model			Protocol		
			**β**	***t*-value**	***p*-value**	**β**	***t*-value**	***p*-value**	**β**	***t*-value**	***p*-value**
**FA**	**CGC**	**Before**	0.000	0.031	0.976	0.001	0.208	0.836	0.010	1.430	0.156
		**ComBat**	0.002	0.211	0.834	0.001	0.240	0.811	0.010	1.347	0.181
		**TS-GLM**	0.000	0.029	0.977	0.000	0.061	0.951	0.010	1.345	0.181
	**CGH**	**Before**	0.020	1.790	0.079	0.016	2.269	0.026	0.011	1.382	0.170
		**ComBat**	0.022	2.067	0.043	0.016	2.735	0.008	0.009	1.209	0.229
		**TS-GLM**	0.020	1.845	0.070	0.017	2.733	0.008	0.011	1.389	0.168
	**Fx-ST**	**Before**	0.032	4.377	0.000	0.027	4.240	0.000	0.018	3.190	0.002
		**ComBat**	0.033	4.728	0.000	0.027	4.779	0.000	0.017	3.195	0.002
		**TS-GLM**	0.032	4.580	0.000	0.031	4.706	0.000	0.019	3.436	0.001
**NDI**	**CGC**	**Before**	−0.015	−1.193	0.238	−0.011	−2.148	0.035	−0.008	−0.882	0.379
		**ComBat**	−0.014	−1.078	0.286	−0.011	−2.482	0.015	−0.005	−0.811	0.419
		**TS-GLM**	−0.015	−1.084	0.283	−0.014	−2.977	0.004	−0.007	−1.063	0.290
	**CGH**	**Before**	−0.030	−2.033	0.047	−0.027	−3.660	0.000	−0.020	−1.825	0.071
		**ComBat**	−0.029	−1.964	0.055	−0.026	−4.338	0.000	−0.015	−2.234	0.028
		**TS-GLM**	−0.029	−2.116	0.039	−0.033	−4.580	0.000	−0.016	−2.205	0.030
	**Fx-ST**	**Before**	−0.008	−0.563	0.576	−0.006	−0.989	0.326	−0.004	−0.301	0.764
		**ComBat**	−0.007	−0.490	0.626	−0.005	−0.938	0.351	0.001	0.119	0.905
		**TS-GLM**	−0.007	−0.516	0.608	−0.010	−1.543	0.127	−0.002	−0.228	0.820

Abbreviations: ComBat, combined association test; TS-GLM, traveling subject-general linear model; FA, fractional anisotropy; MD, mean diffusivity; NDI, neurite density index; ISOVF, isotropic volume fraction; ODI, orientation dispersion index; CGC, cingulum cingulate gyrus; CGH, cingulum hippocampus; Fx-ST, fornix stria terminalis.

© 2023. Saito Y et al. Published online at http://www.aginganddisease.org/EN/10.14336/AD.2023.1020
